# Differential expression of neuropeptide F in the digestive organs of female freshwater prawn, *Macrobrachium rosenbergii*, during the ovarian cycle

**DOI:** 10.1007/s00441-024-03893-8

**Published:** 2024-04-09

**Authors:** Warinthip Vetkama, Ruchanok Tinikul, Prasert Sobhon, Yotsawan Tinikul

**Affiliations:** 1 Department of Anatomy, Faculty of Science, Mahidol University, 272 Rama VI Road, Ratchathewi District, Bangkok 10400, Thailand; 2 Department of Biochemistry and Center for Excellence in Protein and Enzyme Technology, Faculty of Science, Mahidol University, Bangkok 10400, Thailand

**Keywords:** *Macrobrachium rosenbergii*, Female prawn, Neuropeptide F, Digestive organs, Ovarian cycle

## Abstract

Neuropeptide F is a key hormone that controls feeding in invertebrates, including decapod crustaceans. We investigated the differential expression of *Macrobrachium rosenbergii* neuropeptide F (MrNPF) in the digestive organs of female prawns, *M. rosenbergii*, during the ovarian cycle. By using RT-qPCR, the expression of MrNPF mRNA in the esophagus (ESO), cardia (CD), and pylorus (PY) of the foregut (FG) gradually increased from stage II and peaked at stage III. In the midgut (MG), hindgut (HG), and hepatopancreas (HP), MrNPF mRNA increased from stage I, reaching a maximal level at stage II, and declined by about half at stages III and IV (*P* < 0.05). In the ESO, CD, and PY, strong MrNPF-immunoreactivities were seen in the epithelium, muscle, and lamina propria. Intense MrNPF-ir was found in the MG cells and the muscular layer. In the HG, MrNPF-ir was detected in the epithelium of the villi and gland regions, while MrNPF-ir was also more intense in the F-, R-, and B-cells in the HP. However, we found little colocalization between the MrNPF and PGP9.5/ChAT in digestive tissues, implying that most of the positive cells might not be neurons but could be digestive tract-associated endocrine cells that produce and secrete MrNPF to control digestive organ functions in feeding and utilizing feed. Taken together, our first findings indicated that MrNPF was differentially expressed in digestive organs in correlation with the ovarian cycle, suggesting an important link between MrNPF, the physiology of various digestive organs in feeding, and possibly ovarian maturation in female *M*. *rosenbergii*.

## Introduction

The freshwater prawn, *Macrobrachium rosenbergii*, is a highly valuable aquatic species that is cultured worldwide, including in Thailand (New and Nair 2012; Tinikul et al. 2022a, b). Several neurohormones produced and released by the X-organ-sinus gland complex (XO-SG) in the eyestalks are involved in controlling ovarian development in female prawns (Jayasankar et al. 2020), and this may be linked to the nutritional status of the prawns. Feeding and food consumption are crucial physiological processes that female decapod crustaceans require in order to have enough nutrients and energy to promote vitellogenesis and ovarian development (Tinikul et al. 2017; Griffen 2018). In turn, these processes are highly regulated by humoral controls that may also be involved with neuropeptide F (NPF), which directly regulates metabolism and feeding (Nässel and Wegener 2011; Fadda et al. 2019).

Neuropeptide F (NPF) is the invertebrate ortholog of NPY (Li et al. 2018) with high structural similarity, differing only in having a phenylalanine (F) at the C-terminus instead of a tyrosine (Y) (Maule et al. 1992; de Jong-Brink et al. 2001; Fadda et al. 2019). Both neuropeptides belong to the family of FMRFamide-like peptides (FLPs) but with a significant difference in that the former has the C-terminal motif-GRPRFamide and tyrosine residues at positions 10 and 17 from their C-termini (Nässel and Wegener 2011; Christie et al. 2011; Thongrod et al. 2017). Furthermore, NPF has similar physiological functions as NPY in the regulation of food intake and energy homeostasis (Nässel and Wegener 2011; Fadda et al. 2019). There have been reports of NPFs or FLPs being present and playing a role in controlling the digestive organs of insects and crustaceans. In the fruit fly, *Drosophila melanogaster*, NPF-immunoreactivity (-ir) was found in enteroendocrine cells (EECs) of the midgut (MG), where it is co-expressed with other neuropeptides, such as tachykinin and neuropeptide-like precursor 2 (Hung et al. 2020).

NPF expression was observed in the proximal and central parts of the MG of *D. melanogaster* (Veenstra et al. 2008; Nässel 2018; Hung et al. 2020; Yoshinari et al. 2021; Kruangkum et al. 2022). In *D*. *melanogaster*, NPF, a midgut-derived hormone, operates as a sugar-responsive and incretin-like hormone, suggesting that NPF in the MG may play a key role in sugar-dependent metabolic regulation (Yoshinari et al. 2021). In female *Drosophila*, the NPF substantially accumulates in EECs in the middle region of the MG and is released upon mating in response to sex peptide (SP)-dependent signaling (Ameku et al. 2018). NPF belongs to the FMRFamide family. FMRFamide-like ir (FLI-ir) was found in foregut cells (FG cells), endocrine-like cells in the stomach caecae, MG, and hindgut (HG) of male and female locusts, *Locusta migratoria*. There were several endocrine-like FLI-ir cells detected in the MG, implying that FLI may be involved in regulating FG contraction (Hill and Orchard 2003). Additionally, FMRFamide increased muscle contraction of isolated FG in the locust *Schistocerca gregaria* and regulates the action of proctolin and serotonin on the FG (Banner and Osborne 1989). FLI has been found in insect MG endocrine-like cells, including *D. melanogaster* (McCormick and Nichols 1993), *L. migratoria* (Lange 2001), and *Manduca sexta* (Zitnan et al. 1995). The presence of a large number of FLI endocrine-like cells in the MG suggests that the MG functions as an endocrine gland in insects (Orchard et al. 2001; Hill and Orchard 2003).

In addition to the existence and importance of NPF and FMRFamide in controlling digestion in insect species, there have been reports of their presence in crustacean digestive organs. Strong *NPF-I* transcripts were found in the nervous system of penaeid shrimp *Litopenaeus vannamei* and *Melicertus marginatus*, and some expression was detected in MG samples. Notably, a diet supplemented with NPF-I increased food intake and growth, implying that NPF in this shrimp species has orexigenic activity (Christie et al. 2011). In *Scylla paramamosain*, *NPF-II* expression was shown by RT-PCR to be high in the stomach and MG, but low in the hepatopancreas (HP) (Bao et al. 2015). In *Scylla olivacea*, *NPF-II* mRNA expression was detected in the intestine, but not in the stomach or HP, indicating that this isoform of NPF may have a specialized role in the CNS-intestinal axis (Kruangkum et al. 2022). In male prawn *M. rosenbergii*, MrNPF was shown by RT-PCR to be strong in the digestive organs, including the MG and HG, implying its important role in the control of feeding and digestion (Thongrod et al. 2017). In view of the possibility that there may be a correlation between feeding and nutritional status with ovarian development, the expression levels of MrNPF and its anatomical distribution in various digestive organs during the ovarian maturation cycle in female *M. rosenbergii* must be examined. Thus, our research question is whether MrNPF is differentially expressed and found in various parts of digestive organs of female *M. rosenbergii* during the ovarian cycle.

In the present study, we investigated changes in the levels, differential expression, and distribution of MrNPF in the digestive organs of mature *M. rosenbergii* females during the ovarian cycle. We further examined whether NPF is expressed in neurons that may reside in the digestive tract by colocalizing MrNPF with the two neuronal markers, including protein gene product 9.5 (PGP 9.5/UCHL-1) and choline acetyltransferase (ChAT), which is important to understand the possible mode of action of NPF to control digestive cell activities. The findings of this study shed important light on the differential expression of MrNPF in the digestive organs and its correlation with feeding and ovarian development in *M. rosenbergii* females. The knowledge acquired provides the impetus for further research into the possible role of NPF in the regulation of the feeding and other physiological processes of the digestive tract of this commercially important crustacean species.

## Materials and methods

### Experimental animals and acclimatization

Mature *M. rosenbergii* females weighing 31.1 ± 1.5 g were obtained from local markets, Ratchaburi and Ayutthaya Provinces, in Thailand. The prawns were cultured in fiberglass tanks, each measured 1.50 m in diameter and filled with freshwater at a depth of 0.80 m with continuous aeration. At least one-third of the water was changed every 2 days. The water temperature was kept at 27–28 °C, while the pH was set between 7.0 and 8.5. The prawns were fed commercial meal pellets (Charoen Pokphand Group, Thailand) twice a day. To prevent cannibalism, five plastic cages were placed in each tank for molting animals to hide in. The prawns were acclimatized to a photoperiod of 12:12 h light–dark cycle, at least for 1 week before starting the experiments.

### Anatomical nomenclature of digestive organs and classification of ovarian stages

We adopted similar conventional names for these digestive organs as used for other *Macrobrachium* species (Ruiz et al. 2020), and other malacostracan crustacean species (Vogt 1996, 2019; Ceccaldi 2006; Štrus et al. 2019). The ovarian cycle in female *M. rosenbergii* was classified into four stages based on ovary size, color, and histology (Meeratana and Sobhon 2007; Tinikul et al. 2023). A histological examination of these ovarian stages was determined using the standards outlined by Meeratana and Sobhon (2007).

### Gross anatomy and histology of the digestive organs

The female prawns (at least *n* = 6–7 animals per stage) were collected; the digestive organs were morphologically defined and photographed. Each female prawn was divided into two halves along the imaginary line connecting the left and right mandibles, as well as at the cephalothorax-abdominal junction. The anterior half containing foregut (FG) was further dissected for the esophagus (ESO), esophageal gland (ESOG), cardia (CD), pylorus (PY), and hepatopancreas (HP), whereas the posterior half was dissected for the midgut (MG) and hindgut (HG), respectively. After dissection, the digestive tissues were rinsed in phosphate-buffered saline (PBS). These tissues were cut into smaller pieces and promptly immersed in fresh 4% paraformaldehyde at 4 °C for 12–18 h. The tissues were further dehydrated in increasing concentrations of ethanol, and infiltrated with paraffin using an automated tissue processor. The paraffin-embedded tissue blocks were cut to 5–6-µm thickness and stained with hematoxylin and eosin (H&E). The sections were examined and photographed with a Nikon light microscope and digital camera.

### Expression of MrNPF transcripts in the digestive organs using reverse transcription real-time quantitative PCR (RT-qPCR)

The FG, MG, HG, and HP at various ovarian stages were collected (*n* = at least 20 females/digestive part/ovarian stage), immediately snap-frozen in liquid nitrogen, and preserved at −80 °C. RT-qPCR was carried out in accordance with a previously described protocol (Tinikul et al. 2022a), with some modifications. Total RNA was extracted using the TRIzol™ reagent (Invitrogen, Carlsbad, CA, USA) according to the manufacturer’s instructions. These samples were then treated with chloroform and centrifuged at 12,000 rpm for 10 min at 4 °C. RNA was precipitated with isopropanol by incubating the samples for 15 min at −80 °C before centrifuging it for 10 min at 12,000 rpm at 4 °C. The pellet was washed twice with 70% ethanol (v/v). The dried RNA pellet was resuspended in diethylpyrocarbonate (DEPC)-treated water and kept at −80 °C. RNA contents and quality were determined using 1% w/v agarose gel electrophoresis and spectrophotometry (NanoDrop 1000; Thermo Fisher Scientific, DE, USA). All RNA samples had A260/A280 and A260/A230 ratios that were appropriate for RT-qPCR. The DNase treatment used 800 ng of RNA and 1 unit of DNaseI, with the reaction running in 1 DNase reaction buffer (Promega, Wisconsin, USA). After 30 min of incubation at 37 °C, the reaction was stopped by adding 1 L of DNase stop solution. The samples were then incubated at 65 °C for 10 min to inactivate DNase. Subsequently, about 320 ng of RNA was reverse-transcribed into first-strand cDNA using the ImProm-II III Reverse Transcriptase system (Promega, Madison, WI, USA) and random hexamer primers. Then, qPCR was carried out in a 20 µl reaction system containing 2 µl of cDNA (without dilution from reverse transcription), 10 µl of KAPA SYBR FAST qPCR Master Mix (2) (KAPA Biosystems, USA), and 200 nM of forward and reverse primers. The reactions were run on the CFX96 Touch™ Real-Time PCR Detection system (Bio-Rad Laboratories, USA) under the following thermal cycling conditions: 95 °C for 3 min, 40 cycles of 95 °C for 3 s, 60 °C for 20 s, and 65 °C for 5 s. Melting curve analysis was performed at 95 °C for 5 min, followed by 55 to 95 °C, with continuous fluorescence reading at every 0.5 °C increment to ascertain the specificity of the PCR product. Each qPCR run included a negative control with no cDNA. The qPCR primers used in the present study are listed in Table 1. The relative expression levels of MrNPF transcripts were calculated using the 2^−ΔΔCt^ method (Livak and Schmittgen 2001) to assess the fold-changes in MrNPF transcript abundance in digestive organs compared to stage I values. The relative expression of MrNPF mRNA was normalized to that of the *elongation factor 1 alpha* (*EF-1α*) gene (Tinikul et al. 2022a). Reactions were carried out in triplicate.

**Table 1:** Sequences of primers used for RT-qPCR in female *M. rosenbergii*

**Primer**	**Direction**	**Nucleotide sequence (**5′-3′)
MrNPF-F	Forward	5′ CCAAGTGTGGGCGGCTATTT 3′
MrNPF-R	Reverse	5′ TCACCAGGAGGAACGGCATA 3′
EF-1α-F	Forward	5′ GGTGCTGGACAAGCTGAAGGC 3′
EF-1α-R	Reverse	5′ CGTTCCGGTGATCATGTTCTTGATG 3′

### Immunolocalization of MrNPF in the digestive organs during the ovarian cycle

The presence and differential expression of MrNPF detected through its immunoreactivity (MrNPF-ir) in digestive organs, including FG, MG, HG, and HP, were investigated during ovarian stages (at least *n* = 15 animals per collection). These tissues were fixed with fresh 4% paraformaldehyde in 0.1 M PBS at 4 °C for 12 h. After fixation and paraffin embedding, tissue sections were cut at 5–6-µm thickness, mounted on slides coated with 3-aminopropyl triethoxy-silane solution (Sigma-Aldrich Co., St. Louis, MO, USA), and immunofluorescence method was performed based on the previous descriptions (Tinikul et al. 2022a). The sections were then submerged in 1% glycine in PBS for 15 min, followed by 5 min in PBS. Nonspecific binding was blocked by incubating the sections in a humidified chamber for 2 h in a blocking solution containing 10% normal goat serum (NGS) and 0.4% triton-X in PBS (PBST). The sections were incubated in the primary antiserum, a rabbit polyclonal antibody against MrNPF (anti-MrNPF), diluted 1:400, which was produced and tested for its specificity, as previously described (Thongrod et al. 2017; Tinikul et al. 2022b). Additionally, mouse anti-E-Cadherin (diluted 1:100, Santa Cruz Biotechnology), mouse anti-PGP9.5/UCHL-1 (pan-neuronal marker, diluted 1:100, Santa Cruz Biotechnology,), and mouse anti-Choline acetyltransferase (ChAT) (motor neuron marker, diluted 1:100, Santa Cruz Biotechnology) were used. The tissue sections were incubated in these primary antisera in a blocking solution at 4 °C overnight. Subsequently, they were washed three times with PBST and incubated with the secondary antisera Alexa 488-conjugated goat anti-rabbit IgG or Alexa 568-conjugated goat anti-mouse IgG (Molecular Probes, Eugene, USA) diluted at 1:500 in a blocking solution for 2 h. The cell nuclei were stained by DAPI (Sigma-Aldrich, USA) diluted at 1:1000 in the blocking solution for 10 min. The sections were washed again, mounted in glycerol buffer, and imaged using an Olympus FV1000 confocal laser scanning microscope. This experiment was carried out in triplicate. Negative controls were also performed using anti-MrNPF pre-absorbed with the synthetic MrNPF peptide as the primary antiserum or substituting it with pre-immune rabbit serum, or omitting the primary antisera from the staining. All negative control sections showed very weak or no immunoreactivity.

### Imaging analysis

An Olympus FluoView 1000 laser scanning confocal microscope (Olympus America, Center Valley, PA) was used to detect and photograph the immunohistochemical signal of FG, MG, HG, and HP. For each fluorophore, the tissues were scanned sequentially to acquire individual images for each label, and an overlay image of the three channels for each optical region was obtained. Serial optical sections were collated into a single image plane after projections of imaging stacks were generated. Subsets of the z-stacks of 15–20 optical sections were used to create these projected images. The optical section thicknesses inside a confocal stack were tuned and kept consistent during a preparation. The multi-laser intensities were supplied by Alexa Fluor 488, Alexa Fluor 568, DAPI filter sets, as well as image-processing software. Following that, the images were exported from the Olympus confocal system as TIFF files and downloaded to Photoshop CS software (Adobe Systems Inc., USA) for size, brightness, and contrast adjustments as needed to achieve optimal clarity. ImageJ (NIH, Bethesda, MD, USA, available on the internet: http://rsb.info.nih.gov/ij/) was used to quantify the intensity of fluorescence. Furthermore, negative controls for each fluorochrome were scanned using the same parameters for each tissue.

### Evaluation of the numbers of MrNPF-ir cells in the digestive organs during the ovarian cycle

During the ovarian cycle, the numbers of MrNPF-ir positive cells in the digestive organs were counted and compared using the methods previously described (Tinikul et al. 2022b). The digital pictures were inspected and taken at ×20 and ×40 magnifications. Each image contained a resolution of at least 300 dpi. MrNPF-ir cells were counted per visual field (mm^2^ area) using the Cell Counter tool in ImageJ software (NIH, Bethesda, MD, USA; http://rsb.info.nih.gov/ij/). To compare the number of MrNPF-ir cells in various digestive organs during ovarian stages, at least 20–25 random sections per organ from both positive and negative areas were counted. To increase accuracy of quantification, the counting was done in triplicate.

### Assessment of the fluorescence intensity of MrNPF-ir in the digestive organs during the ovarian cycle

The intensity of MrNPF-ir in numerous digestive organ regions was assessed using ImageJ software (NIH, Bethesda, MD, USA, available on the internet: http://rsb.info.nih.gov/ij/), as previously described (Tinikul et al. 2022b), with some modifications. Digital pictures of the sections at magnifications of ×20 and ×40 were acquired from five randomized sections of each area to be examined. Subsequently, each image had a resolution of 300 dpi and was converted to grayscale (Image/Type/8-bit). Additionally, we reduced the bias by completing and comparing with many controls to reassure that the intensity of MrNPF-ir in every digestive tissue during the ovarian cycle was accurate and trustworthy. All immunohistochemistry processes, including the dilutions of primary and secondary antisera, buffer dilutions, and incubation times, remained unchanged. The confocal conditions, notably the panel for image collection and laser channel modification, were monitored on a regular basis.

### Statistical analyses

Data were presented as means ± S.E.M. The data were then analyzed for statistical differences with the SPSS program from Windows software (SPSS Inc., Chicago, IL, USA), using a one-way analysis of variance (ANOVA) and Tukey post hoc test. A probability value less than 0.05 (*P* < 0.05) indicated a significant difference.

## Results

### Gross anatomy and histology of different ovarian stages and digestive organs

**Fig. 1:**
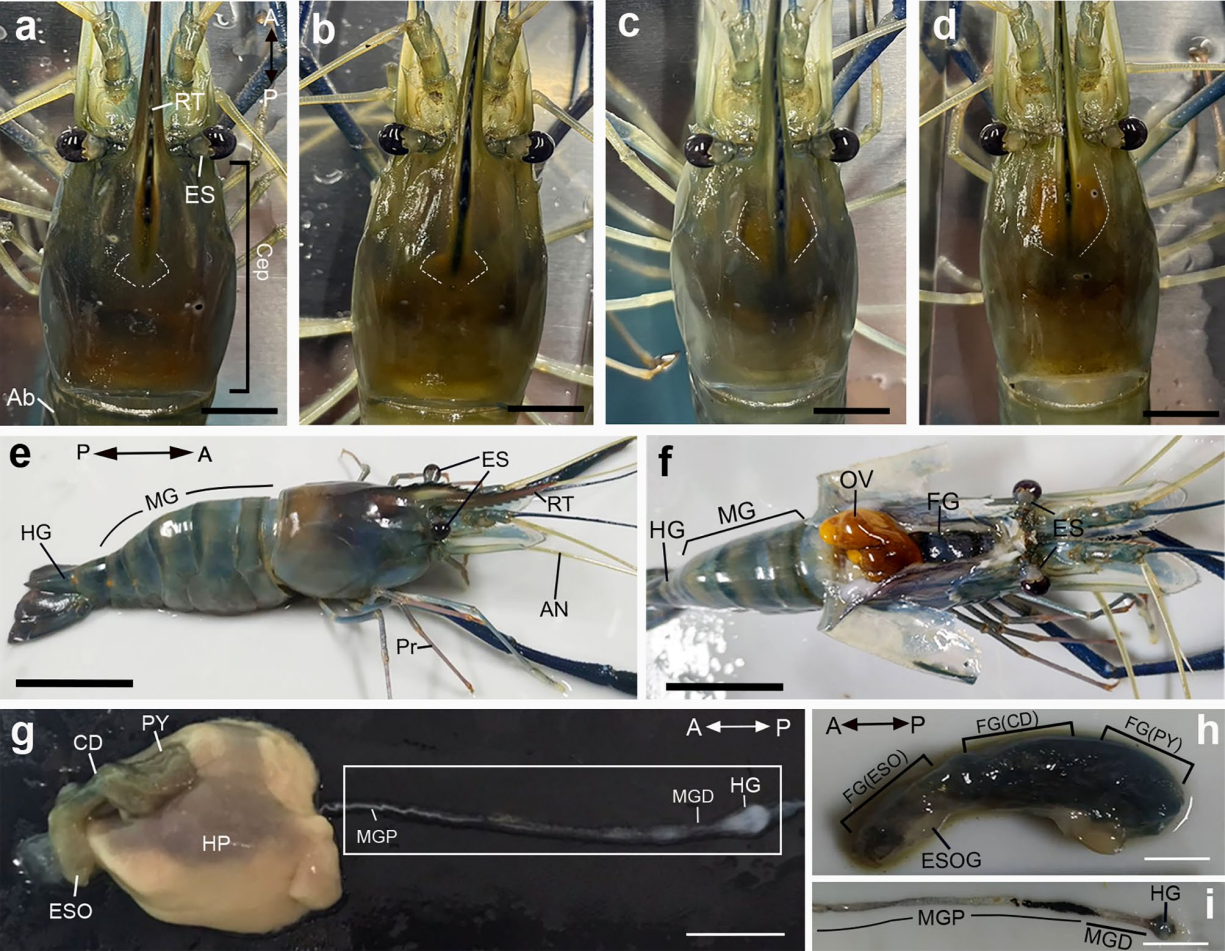
Top views of female prawns, *M. rosenbergii* demonstrating various ovarian stages I (**a**), II (**b**), III (**c**), and IV (**d**), respectively. The dashed lines represent the location of the ovaries. **e**,** f** Morphological organization of digestive organs in female *M. rosenbergii*, lateral (**e**) and top (**f**) views, showing the location of the FG and HP at Cep region, while the MG and HG are in the Ab region. **g** Dissected ESO, CD, and PY parts of the FG, MGP, MGD, and HG. **h** Representative images of dissected ESO, CD, and PY parts. **i** MGP, MGD, and HG parts. The orientation of digestive organs is shown in **a**, **e**, **g**, and **h**. A, anterior; Ab, abdomen; AN, antenna; CD, cardiac; Cep, cephalothorax; ES, eyestalk; ESO, esophagus; ESOG, esophageal gland; FG, foregut; HG, hindgut; HP, hepatopancreases; MG, midgut; MGD, distal part of midgut; MGP, proximal part of midgut; OV, ovary; P, posterior, Pr, periopods; PY, pylorus; RT, rostrum. Scale bar 1 cm (**a**–**d**), 2.5 cm (**e**–**g**), and 0.5 cm (**h**–**i**)

The gross anatomy and histology of the ovaries and digestive organs are given here to assist readers in navigating the anatomical parts related to MrNPF differential expression and distribution (Fig. 1a–i). The top view of ovarian development during the ovarian cycle for a more relevant comprehension in this female prawn is shown in Fig. 1a–d. At stage I, which is the spent phase, only a vestige of the ovary is visible through the carapace (Fig. 1a). At stage II, which is the proliferative phase, a tiny yellow mass of ovary is visible under the carapace (Fig. 1b). The ovary becomes enlarged and turns orange in stage III, which is the premature phase (Fig. 1c). The ovary becomes fully enlarged, extends from the back of the eye to the first abdominal segment, and turns reddish-orange at stage IV, which is the mature phase (Fig. 1d).

The digestive organs of female *M. rosenbergii* can be morphologically categorized into three distinct parts, namely the FG, MG, and HG. The HP is a digestive organ connected to the digestive tract and is included with this system. The FG is comprised of three regions, namely the ESO, CD, and PY (Fig. 1e). The ESO serves as the initial segment of the FG responsible for receiving ingested food from the oral cavity. The CD refers to the central region of the FG, which functions as the recipient of nutrients from the ESO, as depicted in Fig. 1e and f. The posterior portion of the FG, known as the PY, acts as the recipient of food from the CD (Fig. 1g, h). The MG is divided into two distinct parts: the proximal segment (MGP) and the distal segment (MGD). The MG is a cylindrical structure that undergoes a transformation into a bulbous structure at its distal end, which subsequently develops into the HG and anus (Fig. 1g, i).

### Expression levels of MrNPF transcripts in the digestive organs of female *M. rosenbergii* during the ovarian cycle as determined by RT-qPCR

**Fig. 2:**
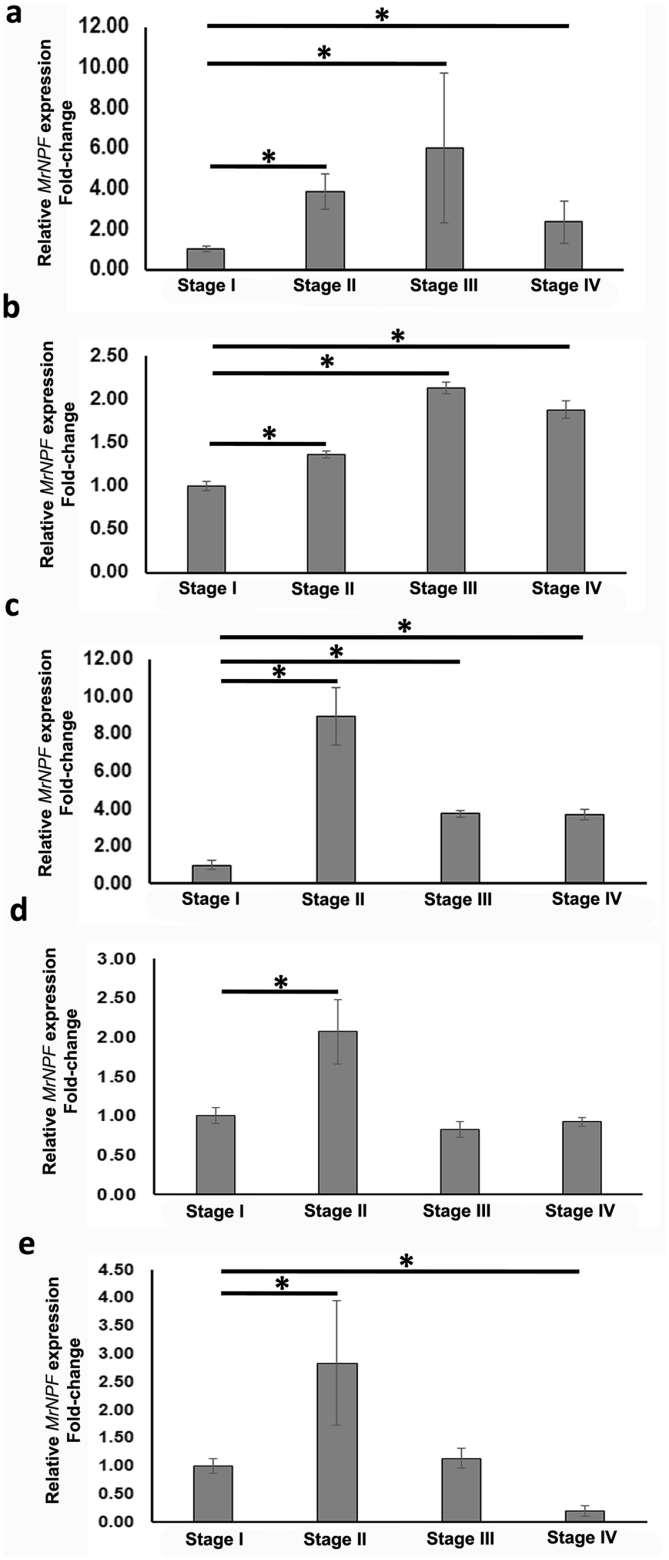
Relative expression of MrNPF mRNA in the ESO (**a**), CD and PY (**b**), MG (**c**), HG (**d**), and HP (**e**) during the ovarian cycle of female *M. rosenbergii*, as determined by RT-qPCR (*n* = 20 for each ovarian stage (I–IV)). MrNPF transcript numbers were normalized against the reference gene, *EF-1α.* These histograms demonstrate the highest levels of MrNPF mRNA at stages II (MG, HG, and HP) and III (ESO and CD/PY). Asterisks indicate significant differences (*P* < 0.05), compared with stage I. Each measurement is expressed as mean ± SEM

By RT-qPCR, the expression of MrNPF levels in the ESO was shown to gradually increase from stage I to stage II (fourfold), peaked at stage III (6.1-fold), and declined at stage IV (2.2-fold) (Fig. 2a). The mRNA expression levels of the MrNPF from these three stages were significantly higher than those at stage I (*P* < 0.05) (Fig. 2a). In the CD and PY, the expression patterns of MrNPF mRNA appeared to be similar to those in the ESO. Specifically, MrNPF mRNA increased from stage I to stage II (1.5-folds), and reached higher levels at stage III (2.2-folds), and slightly decreased at stage IV (1.8-folds). These MrNPF mRNA levels from stages II-IV were significantly increased, compared with stage I (*P* < 0.05) (Fig. 2b). In the MG, The mRNA expression levels of the MrNPF increased from stage I, reaching a maximal level at stage II (9-fold), and declined to about half at stages III and IV (4-fold). These MrNPF mRNA levels from these stages were significantly different compared with stage I (*P* < 0.05) (Fig. 2c). Notably, the MrNPF level in the MG at stage II was higher than those detected in other parts of the digestive tissues (Fig. 2c). Similarly, the expression level of MrNPF mRNA in the HG was highest at stage II (2.2-fold), and decreased at the latter two late stages. MrNPF mRNA levels in the HG were comparable with those detected in the CD and PY (Fig. 2d). Comparatively, the fold-changes in MrNPF expression levels from the MG at ovarian stage II were about 2 and 6 times higher than those detected in the ESO and CD/PY, respectively. In the HP, the pattern of MrNPF expression was similar to that detected in the MG and HG parts, with the highest MrNPF level detected at stage II (3-fold), and the level gradually decreased at late stages by about 1.3-fold, respectively (Fig. 2e).

### Existence and distribution of MrNPF-ir in the digestive organs during the ovarian cycle

#### The existence and distribution of MrNPF-ir in the FG

**Fig. 3:**
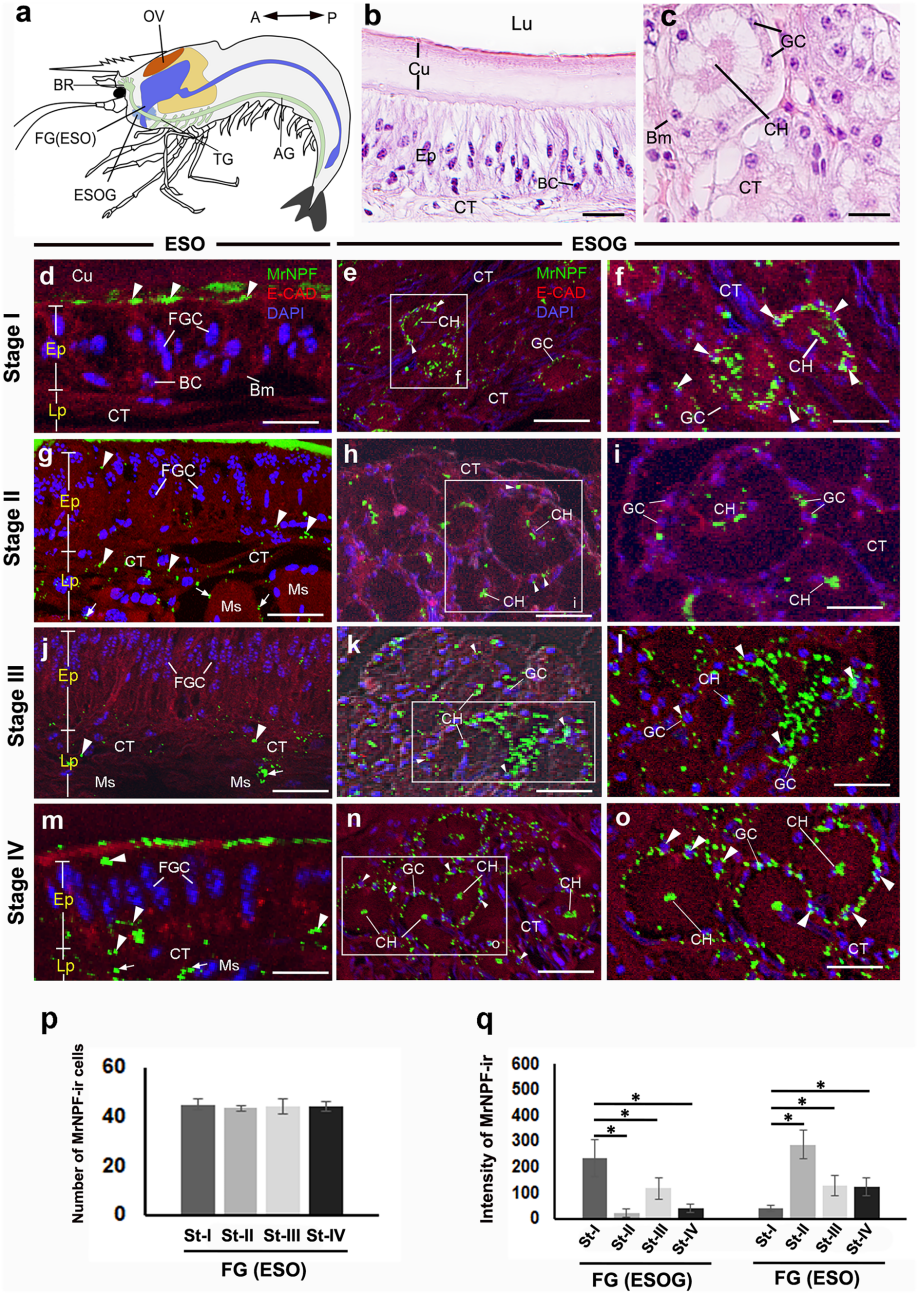
MrNPF immunolabelling (green) in the ESO and ESOG parts of the FG of female *M. rosenbergii* during ovarian stages I–IV (at least 15 female prawns). E-Cadherin-ir was stained (red), while nuclei were counterstained with DAPI (blue). **a** Schematic representation of the location of ESO and ESOG. The orientation is given on the top right of **a**. **b**, **c** Histological images of the ESO (**b**) and ESOG (**c**). **d**, **g**, **j**, and **m** MrNPF-ir in the ESO at ovarian stages I–IV, respectively. An intense MrNPF-ir is observed in FGCs, CT, and Ms (white arrows). **e**, **f**, **h**, **i**, **k**, **l**, **n**, and **o** MrNPF-ir in the ESOG at stages I-IV, which strong MrNPF-ir is seen in the GC and CH parts (white arrowheads). **p**, **q** Histograms representing the number of MrNPF-ir cells in the ESO (**p**), and fluorescence intensity (**q**) in the ESO and ESOG during the ovarian cycle. All data are presented as the mean ± SEM. Asterisks indicate significant differences (*P* < 0.05). AG, abdominal ganglion; BC, basal cell; Bm, basement membrane; BR, brain; CH, central lumen; CT, connective tissue; Cu, cuticle; Ep, epithelium; ESO, esophagus; ESOG, esophageal gland; FGC, foregut cell; GC, gland cell; Lp, lamina propria; Lu, lumen; Ms, muscle; OV, ovary; St-I to St-IV, stage I to stage IV; TG, thoracic ganglion. Scale bars 100 µm (**e**, **h**, **k**, **n**) and 50 µm (**b**, **c**, **d**, **f**, **g**, **i**, **j**, **l**, **m**,** o**)

The location and histological features of the ESO and ESOG are shown (Fig. 3a–c). The epithelium (Ep) of ESO contains two types of cells, including foregut cells (FGCs) and basal cells (BCs) (Fig. 3b). The ESOG is located beneath the tegument of the mouthparts, and its secretory cells (GC) are arranged around a central lumen (CH) that conveys the secretions from the glands (Fig. 3c). Strong MrNPF-ir was detected extensively in the ESO and ESOG during the ovarian cycle. In the ESO, intense MrNPF-ir was detected in the apical and basal regions of some FGCs of Ep. Additionally, the apical surface of FGCs, the muscle (Ms), and the connective tissue (CT) in the lamina propria (Lp) layer also displayed strong immunoreactivity (Fig. 3d, g, j, m). In the ESOG, MrNPF-ir was present extensively in GCs, and some strong immunoreactivity was seen in the CH of several acini (Fig. 3e, f, h, i, k, l, n, and o). The number of MrNPF-ir positive cells in the ESO was approximately 40–45 cells in each ovarian stage, but their numbers showed no significant difference during the ovarian cycle (*P* > 0.05) (Fig. 3p). The intensity of MrNPF-ir in the ESO was highest at stage II, compared with the late stages (*P* < 0.05), while the intensity in the ESOG was shown to be higher at the early ovarian stages, but decreased at the late stages (*P* < 0.05) (Fig. 3q).

**Fig. 4:**
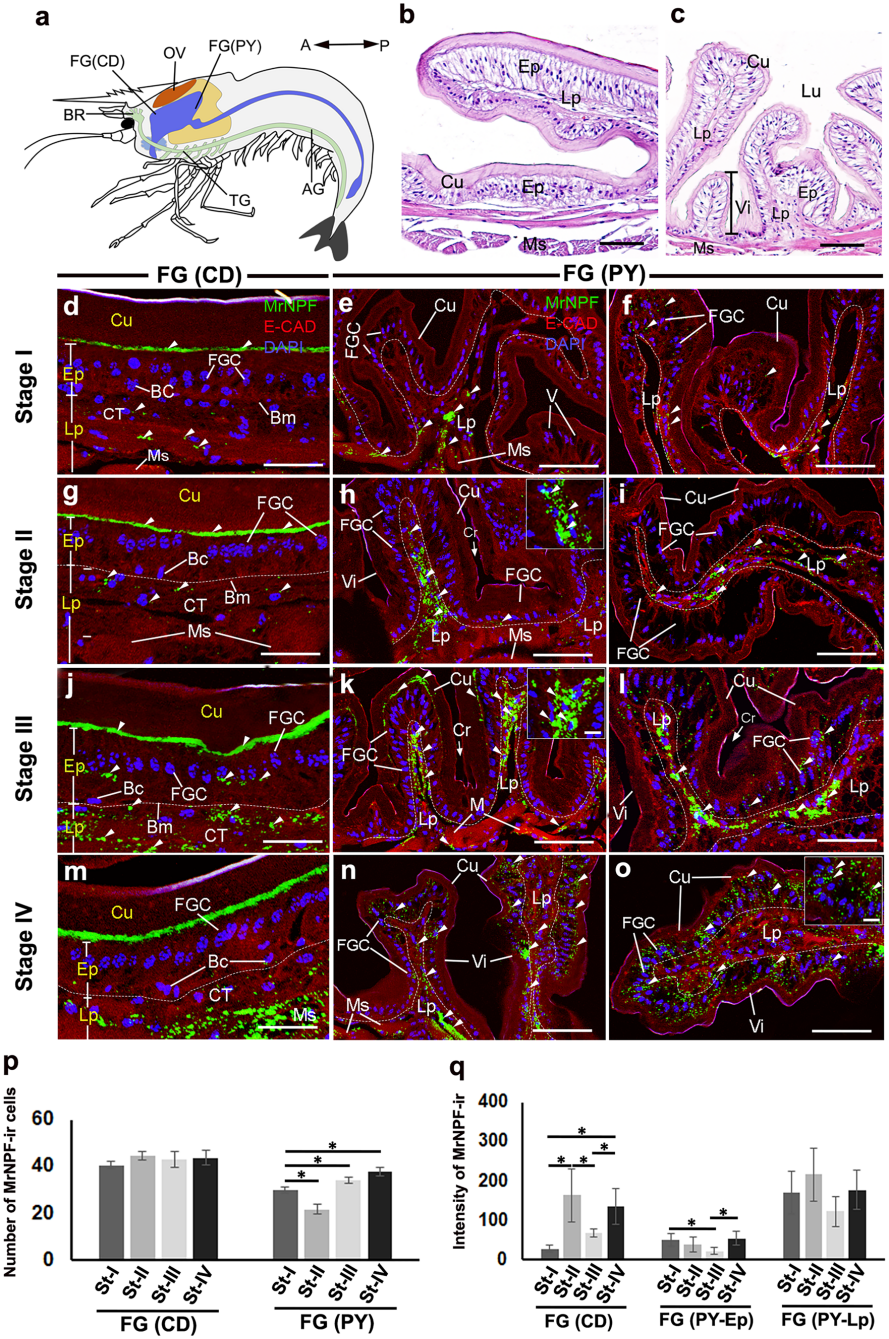
MrNPF immunolabelling (green) in the CD and PY parts of the FG during ovarian stages I–IV of female *M. rosenbergii* (at least *n* = 15 female prawns). **a** Schematic representation of the location of CD and PY. **b**, **c** Histological images of the CD (**b**) and PY (**c**). **d**, **g**, **j**, and **m** Intense MrNPF-ir is seen in Ep, Lp (white arrowheads), and lower Cu areas (white arrowheads) of the CD at stages I–IV (**d**, **g**, **j**, and **m**), respectively. **e**, **f**, **h**, **i**, **k**, **l**, **n**, and **o** MrNPF-ir in the PY in stages I–IV, and intense MrNPF-ir is observed in the Ep, Lp, and Ms layers of the PY part (white arrowheads). **p**, **q** Histograms representing the number of MrNPF-ir cells (**p**) and fluorescence intensity (**q**) in the CD and PY during ovarian maturation stages. All data are presented as the mean ± SEM. Asterisks indicate significant differences (*P* < 0.05). AG, abdominal ganglion; BC, basal cell; Bm, basement membrane; BR, brain; CD, cardia; Cu cuticle; FGC, foregut cell; FG, foregut; FG (PY-Ep), epithelial layer of pylorus; FG (PY-Lp), lamina propria layer of pylorus; Lp, lamina propria; Ms, muscle; OV, ovary; PY, pylorus; TG, thoracic ganglion; Vi, villi. Scale bars 100 µm (**b**, **c**), 50 µm (**d**–**o**), and 25 µm (inset **h**, **k**, **o**)

The location and the histology of the CD and PY are demonstrated (Fig. 4a–c). In the CD, intense MrNPF-ir was observed in the apical and basal areas of the FGCs (Fig. 4d, g, j, and m). Notably, many varicose MrNPF-ir fibers in the Lp was also strongly labeled in the PY (Fig. 4e, f, h, i, k, l, n, o). In the deep part of the Lp, many strong immunoreactive fibers were close to the Ep cells in the intestinal gland crypts. The number of MrNPF-ir cells in the CD was approximately 40–46 cells at each stage, and the number of MrNPF-ir cells in this region was not significantly different during the ovarian cycle (*P* > 0.05) (Fig. 4p). In the PY, the number of MrNPF-ir cells increased from stage III (approximately 36 cells) and reached its highest number at stage IV (approximately 42 cells), but the least number of MrNPF-ir cells was detected at stage II (approximately 22 cells) (*P* < 0.05) (Fig. 4p). The fluorescence intensities of MrNPF-ir in the CD increased at stage II, but declined at stages III and IV (*P* < 0.05), while the intensity of MrNPF-ir in the Ep part of PY mostly appeared to be lower than that found in the CD (*P* < 0.05) (Fig. 4q). The intensity of MrNPF-ir in the Lp part of PY appeared to be highest at stage II, and the intensity was significantly higher in MrNPF-ir in the Lp part than in the Ep part of PY during the ovarian cycle (Fig. 4q).

#### The expression and distribution of MrNPF-ir in the MG

**Fig. 5:**
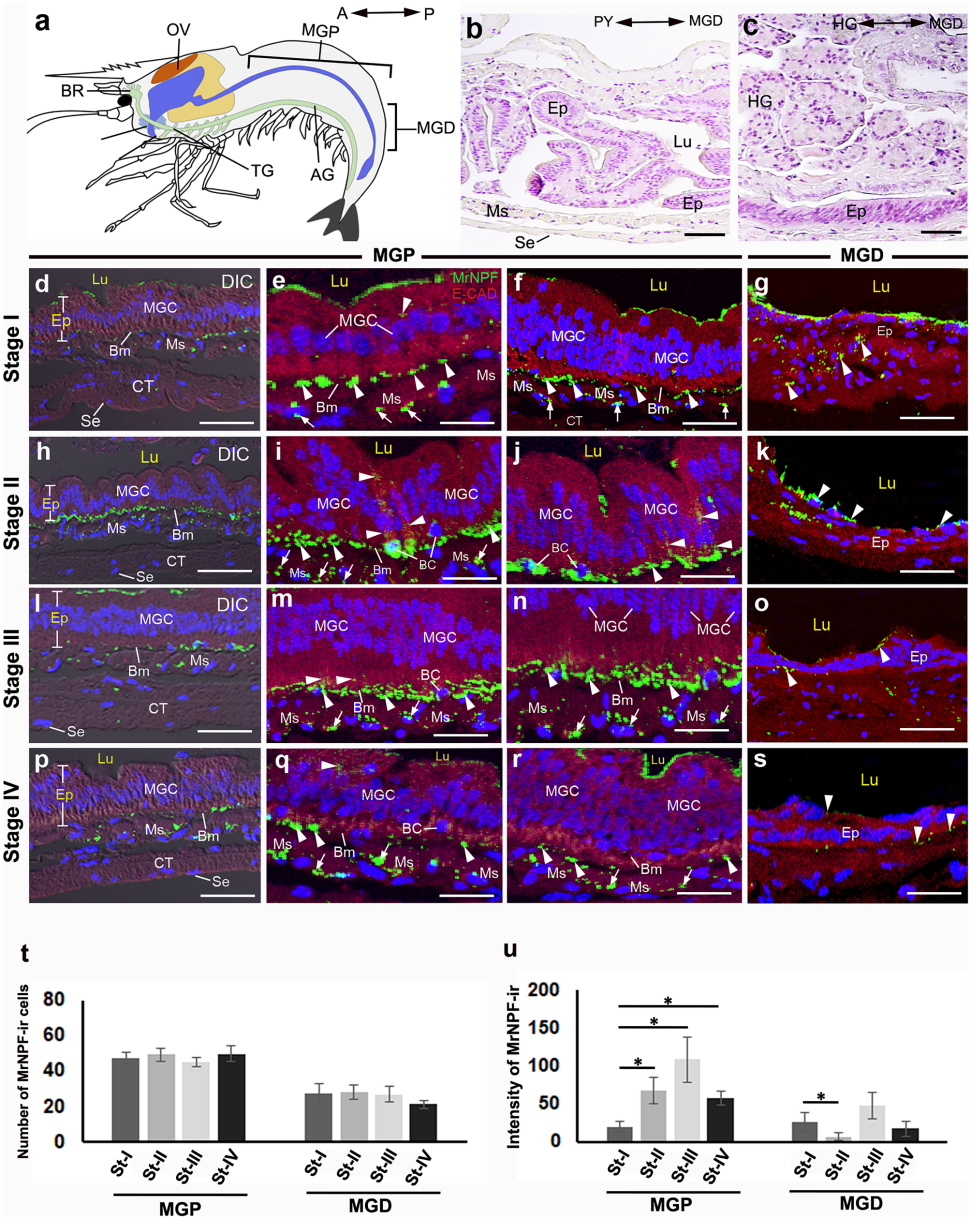
MrNPF immunolabelling (green) in the MG during ovarian stages I–IV of female *M. rosenbergii* (at least *n* = 15 female prawns). **a** Schematic representation of the location of MGP and MGD parts. The orientation is given on the top right (**a**). **b**, **c** The histology of MGP (**b**) and MGD (**c**) structures. **d**,** h**, **l**, and **p** Images showing strong MrNPF-ir in the MGP in stages I–IV. The figures **d**,** h**,** l**, and** p** are merged with the differential interference contrast (DIC) images. **e**, **f**, **i**, **j**, **m**, **n**, **q**, and **r** Representative images demonstrating MrNPF-ir in MGP at stages I–IV. Strong MrNPF-ir is shown in the MGCs, clearly visible in the BCs, and in the Bm (white arrowheads). MrNPF-ir is also found in the Ms layer (white arrows). **g**, **k**, **o**, and **s** The Ep and Lp layers (white arrowheads) in the MGD display immunoreactivity during ovarian stages I–IV. **t**–**u** Histograms representing the number of MrNPF-ir cells (**t**) and fluorescence intensity (**u**) in the MGP and MGD parts during the ovarian cycle. All data are presented as the mean ± SEM. Asterisks indicate significant differences (*P* < 0.05). AG, abdominal ganglion; BC, basal cell; Bm, basement membrane; BR, brain; CT, connective tissue; Ep, epithelium; Lp, lamina propria; Lu, lumen; MGC, midgut cell; MGD, distal part of midgut; MGP, proximal part of midgut; Ms, muscle; OV, ovary; Se, serosa. Scale bars 100 µm (**b**, **c**, **d**, **h**, **l**, and **p**) and 50 µm (**e**–**s**)

The position and the histology of the MG are shown in Fig. 5a–c. In the MGP, MG cells (MGCs) showed strong MrNPF-ir (Fig. 5d–f, h–j, l–n, and p–r). At the basal region, intense MrNPF-ir was seen in the BC, and extensive immunoreactivity was observed in the junction between the Ep and Ms layers. It is noteworthy that the circular Ms layer was strongly MrNPF immunoreactive (Fig. 5e, i, m, and q). However, MrNPF-ir was seen in the Ep in the MGD (Fig. 5g, k, o, and s). Approximately 45–50 MrNPF-ir cells were detected in the MGP at each ovarian stage, and there was no significant difference in this number throughout the ovarian cycle (*P* > 0.05) (Fig. 5t). Notably, at each ovarian stage, the number of MrNPF-ir cells in the MGD was approximately two times fewer (about 20–25 cells) than that of the MGD (Fig. 5t). The MGP at stage III had the highest MrNPF-ir intensity, whereas stage I had the lowest intensity (Fig. 5u). The intensity of MrNPF-ir in the MGD was highest at stage III, but lowest intensity was detected at stage II (Fig. 5u).

### The presence and distribution of MrNPF-ir in the HG

**Fig. 6:**
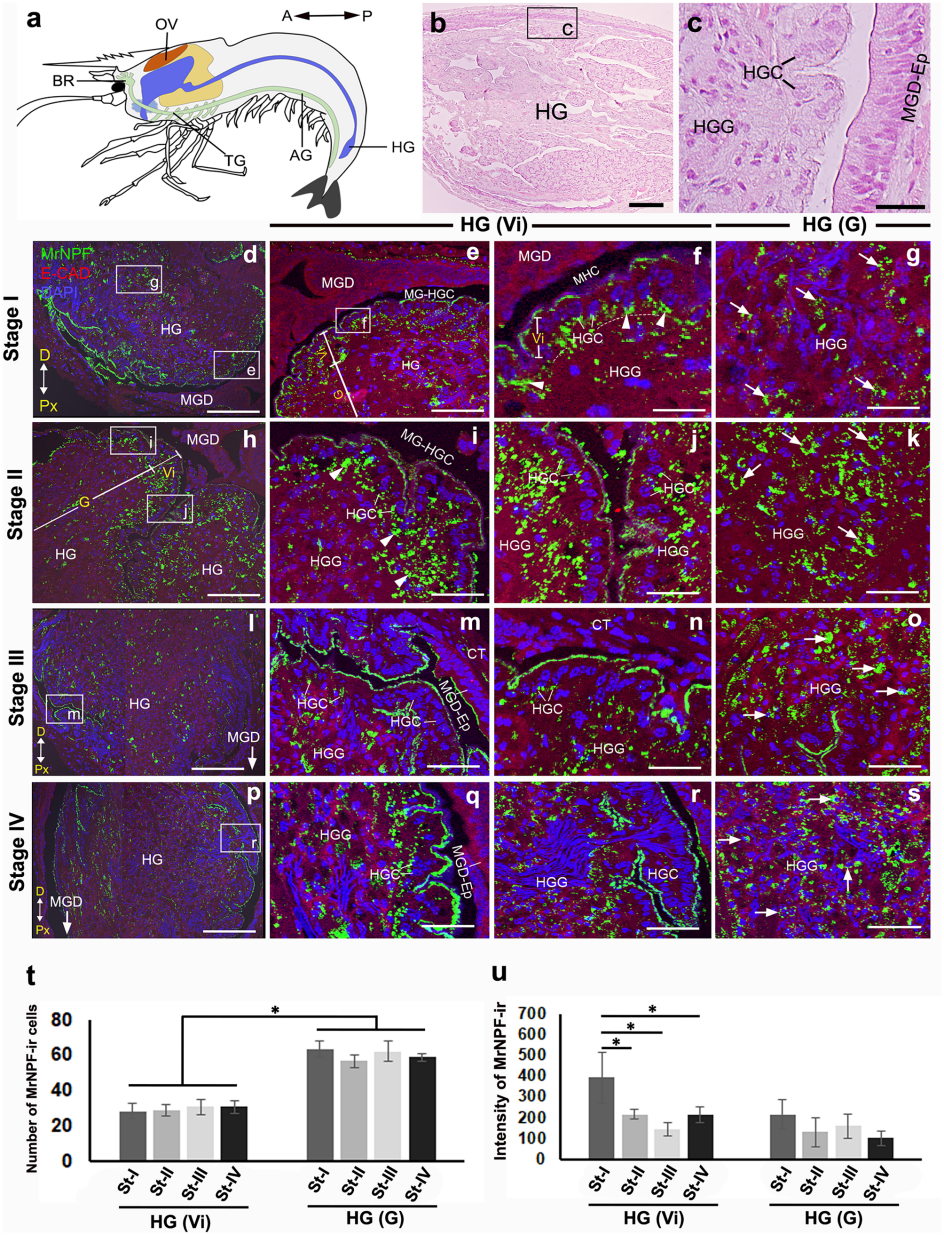
MrNPF immunolabelling (green) in the HG during ovarian stages I–IV of female *M. rosenbergii* (at least *n* = 15 female prawns). **a** Schematic representation of the location of the Vi and G parts with the orientation is given on the top right. **b**, **c** Histological images showing the Vi (HGCs) (**b**) and HGG (**c**) structures. **d**, **h**, **i**, and **p** Images exhibiting MrNPF-ir in the Vi and G parts of HG in stages I–IV, respectively. The figures **d**, **h**, **l**, and **p** are merged with DIC images. Strong MrNPF-ir is detected in both HGCs of the Vi (white arrowheads) and HGG in the G parts (white arrows). **f**, **j**, **n**, and **r** Images showing MrNPF-ir in the Vi part, and strong MrNPF-ir is shown in the apical surface of HGCs at stages I-IV. **g**, **k**, **o**, and **s** Intense MrNPF-ir in the G part at stages I–IV, respectively. **t**–**u** Histograms showing the number of MrNPF-ir cells (**t**) and fluorescence intensity (**u**) in both Vi and G parts of HG during the ovarian cycle. All data are presented as the mean ± SEM. Asterisks indicate significant differences (*P* < 0.05). A, anterior; D, distal; G, gland part; HG, hindgut; HGC, hindgut cell; HGG, hindgut gland; MGD, midgut distal part; MGD-Ep, midgut distal part (epithelium); MG-HGC, midgut-hindgut channel; P, posterior; Px, proximal; Vi, villi part. Scale bars 100 µm (**b**, **d**, **h**, **l**, and **n**) and 50 µm (**e**, **f**, **g**, **i**, **o**, **k**, **m**, **n**, **o**, **q**, **r**, **s**)

The location and the histology of the HG are demonstrated (Fig. 6a–c). This gut region has two main parts, including the villi (Vi) and gland (G) parts. Strong MrNPF-ir was detected exclusively in the Vi and G areas during the ovarian cycle (Fig. 6d, h, l, and p). Intense MrNPF-ir was observed in the apical and basal regions of the cytoplasm of Ep cells of Vi (Fig. 6e and f, i and j, m and n, and q–r, arrowheads). In the G region, we detected strong MrNPF-ir in the G cells (Fig. 6g, k, o, and s). In the Vi part of HG, the number of MrNPF-ir cells was approximately 24–30 cells, while there were about 2.7 times more cells (55–62 cells at each ovarian stage) in the G region (*P* < 0.05). However, there were no significant differences in MrNPF-ir cell numbers in each part of the HG during the ovarian cycle (Fig. 6t). The intensity of MrNPF-ir in the Vi part was higher in the early stages, compared with the late stages (*P* < 0.05), whereas the intensity in the G region was shown to be higher at the early stages of ovarian development, when compared with the later stages (*P* > 0.05) (Fig. 6u).

#### The expression and distribution of MrNPF-ir in the HP

**Fig. 7:**
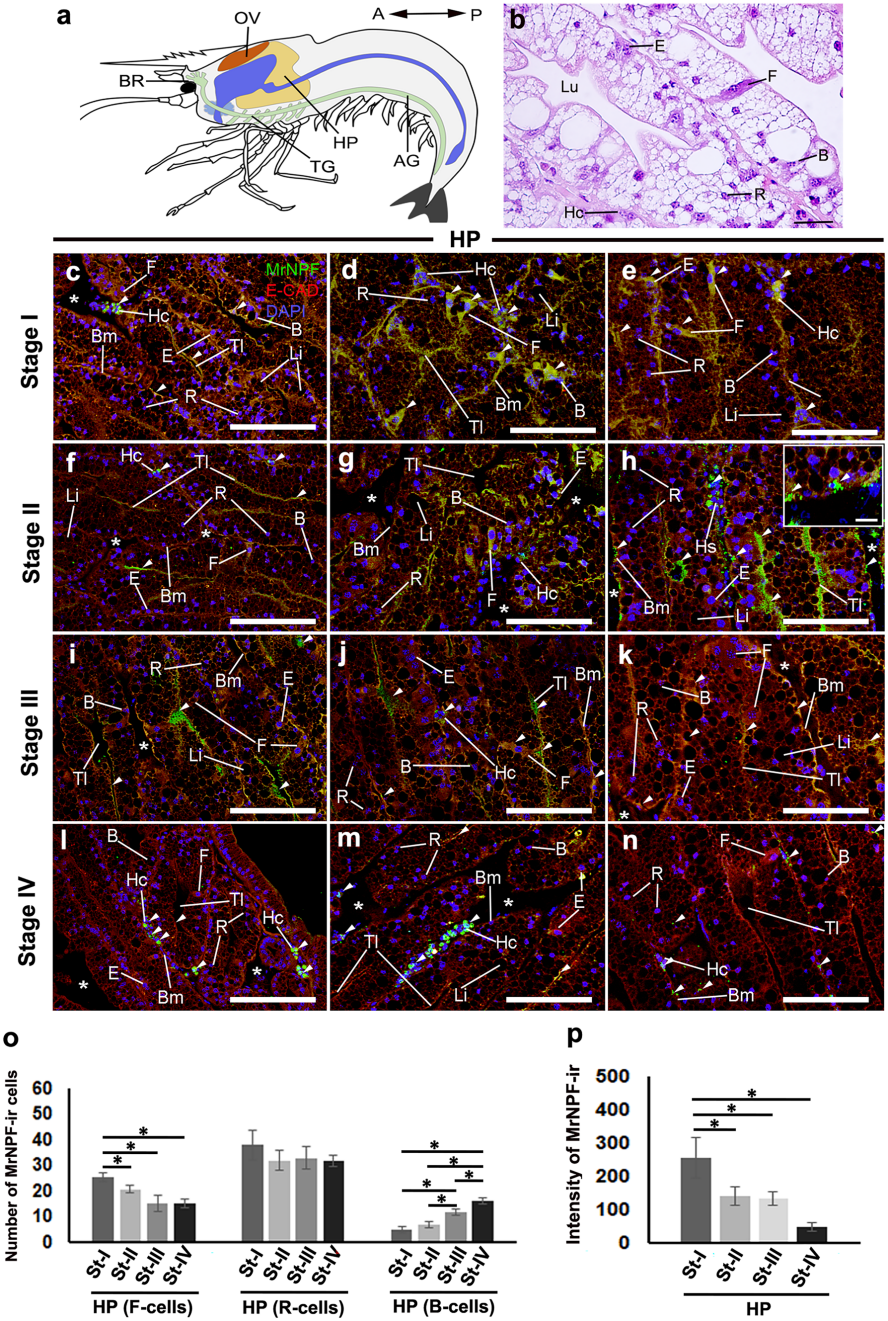
MrNPF (green) immunolabelling in the HP during ovarian stages I–IV of female *M. rosenbergii* (at least *n* = 15 female prawns). **a** Schematic representation of the location of HP. **b** Histological pictures demonstrating several cell types and structures in the HP. **c**–**n** Strong MrNPF-ir is detected in F-, R-, B-cells, and in HC (white arrowheads) at ovarian stages I–IV. The Tl and Bl (white arrowheads) also displayed MrNPF-ir. Notably, strong immunoreactivity is observed in several cell types at stages I and II (**d**, **e**, **g**, and **h**). Strong immunoreactivity is found in the Hc (for example, **c**, **h**, **l**, and **m**). **o**, **p** Histograms representing the number of MrNPF-ir cells (**o**) and fluorescence intensity (**p**) in F-, R-, and B-cells of HP during ovarian maturation. All data are presented as the mean ± SE. Significant *P* values are shown (*P < 0.05). B, blister/vesicular cells; Bm, basement membrane; BR, brain; E, embryonic cells; F, fibrillar cells; Hc, hemocyte; HP, hepatopancreas; Li, lipid droplets; OV, ovary; R, resorptive cells; Tl, tubular lumen; *, interlobular area. Scale bars 100 µm (**b**, **e**, **h**, **k**), 50 µm (**c**, **d**, **f**, **g**, **h**, **i**, **j**, **l**, **m**), and 25 µm (inset of **h**)

The anatomical position and histology of the HP containing several cell types are provided (Fig. 7a and b). We identified the HP cell types in female *M. rosenbergii* based on the characteristics of HP cells in decapod crustaceans, as described previously (Silva et al. 2018; Vogt 2019; Štrus et al. 2019, and Ruiz et al. 2020). Briefly, the HP has several cell types, including fibrillar cells (F-cells), blister/vesicular cells (B-cells), and resorptive cells (R-cells). F-cells possess a sizable and rather extended nucleus, whereas B-cells are characterized as vesicular cells with large supra-nuclear vacuoles, and each cell exhibit a spherical shape with a rounded basal nucleus. The nuclei of the R-cells are spherical and located in the lower part of the cell. The nucleus often had a prism-like form and the cytoplasm contained numerous small vacuoles. Intense MrNPF-ir was observed in the major cell types of HP, including F-, R-, and B-cells. Additionally, MrNPF-ir was seen in the tubular lumen (Tl) and hemocytes (Hc) of HP during the ovarian stages (Fig. 7c–n). MrNPF-ir in F-cells was more prevalent in the early stages (approximately 26 cells), compared with the late stages (*P* < 0.05) (Fig. 7o). As well, there was a decrease in the number of F-cells from stage II to stage IV (about 17, 13, and 12, respectively) (Fig. 7o). Additionally, stage I had the maximum number of MrNPF-ir in R-cells (about 39 cells), while stage IV had the lowest number (around 34 cells), albeit with no significant difference between the two stages (*P* > 0.05) (Fig. 7o). By contrast, the number of MrNPF-ir in B-cells increased considerably from the early stages (approximately 7 cells) to the late stages (about 21 cells) (*P* < 0.05) (Fig. 7o). The early stages of the HP had the highest intensities, whereas stage IV had the lowest intensity of MrNPF-ir (Fig. 7p).

### Colocalization of MrNPF with PGP9.5/UCHL-1 or ChAT in digestive organs

**Fig. 8:**
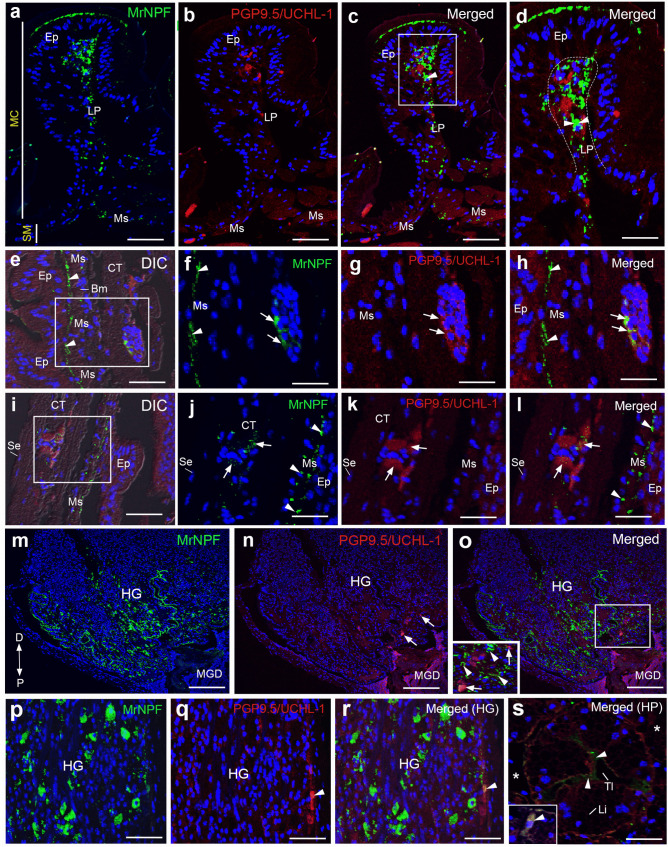
Representative images showing the presence and colocalization of MrNPF (green) and PGP9.5/UCHL-1-like-ir (red) immunolabelling in the FG, MG, HG, and HP of female *M. rosenbergii* (at least *n* = 15 female prawns), while nuclei were counterstained with DAPI (blue). **a**–**d** Representative images demonstrating the expression of MrNPF-ir and PGP9.5/UCHL-1-like-ir, respectively (**a** and **b**). Strong PGP9.5/UCHL-1-like-ir is observed in the Lp (white arrowheads) in the PY. Colocalization of intense MrNPF-ir and PGP9.5/UCHL-1-like-ir in the Lp layer of the PY region is seen (**c** and **d**). **e**, **i** Intense MrNPF-ir and PGP9.5/UCHL-1-like-ir in MG. The figures **e** and **i** are merged with DIC images. **f**, **j** MrNPF-ir is observed in some cells in the Ms (white arrowheads) and the CT layer (white arrows), while strong PGP9.5/UCHL-1-like-ir is expressed in a few cells of the CT layer (white arrows) of MG (**g**, **k**). **h**, **l** Colocalization of a few MrNPF-ir and PGP9.5/UCHL-1-like-ir cells is demonstrated in the CT layer of MG (white arrows). **m**, **n** Images showing MrNPF-ir in Vi and G of HG, whereas intense PGP9.5/UCHL-1-like-ir was detected in the Vi parts of HG (white arrows). **o** Colocalization of a few MrNPF-ir and PGP9.5/UCHL-1-like-ir cells in the Vi is observed (inset in **o**, white arrows shows colocalization, while white arrowheads only exhibit Mr-NPF-ir). **p**, **q** The prevalence of MrNPF-ir in the G part of HG (white arrowhead), while little colocalization of a few MrNPF-ir and PGP9.5/UCHL-1-like-ir cells in the HGG region (white arrowhead) (**r**). **s** Little colocalization of MrNPF-ir and PGP9.5/UCHL-1-like-ir in the HP (white arrowheads), and a few colocalized cells are observed (inset). Bm, basement membrane; CT, connective tissue; Ep, epithelial cell; HG, hindgut; Li, lipid droplets; Lp, lamina propria; MC, mucosa layer; MGD, midgut distal part; Ms, muscle; Se, serosa; SM, submucosa layer; Tl, tubular lumen; *, interlobular area. Scale bars 100 µm (**a**, **b**, **c**,** e**, **i**, **m**, **n**, and **o**), 50 µm (**d**, **f**, **g**, **h**, **j**, **k**, **l**, **p**, **q**, and **r**), and 25 µm (inset of **o**)

**Fig. 9:**
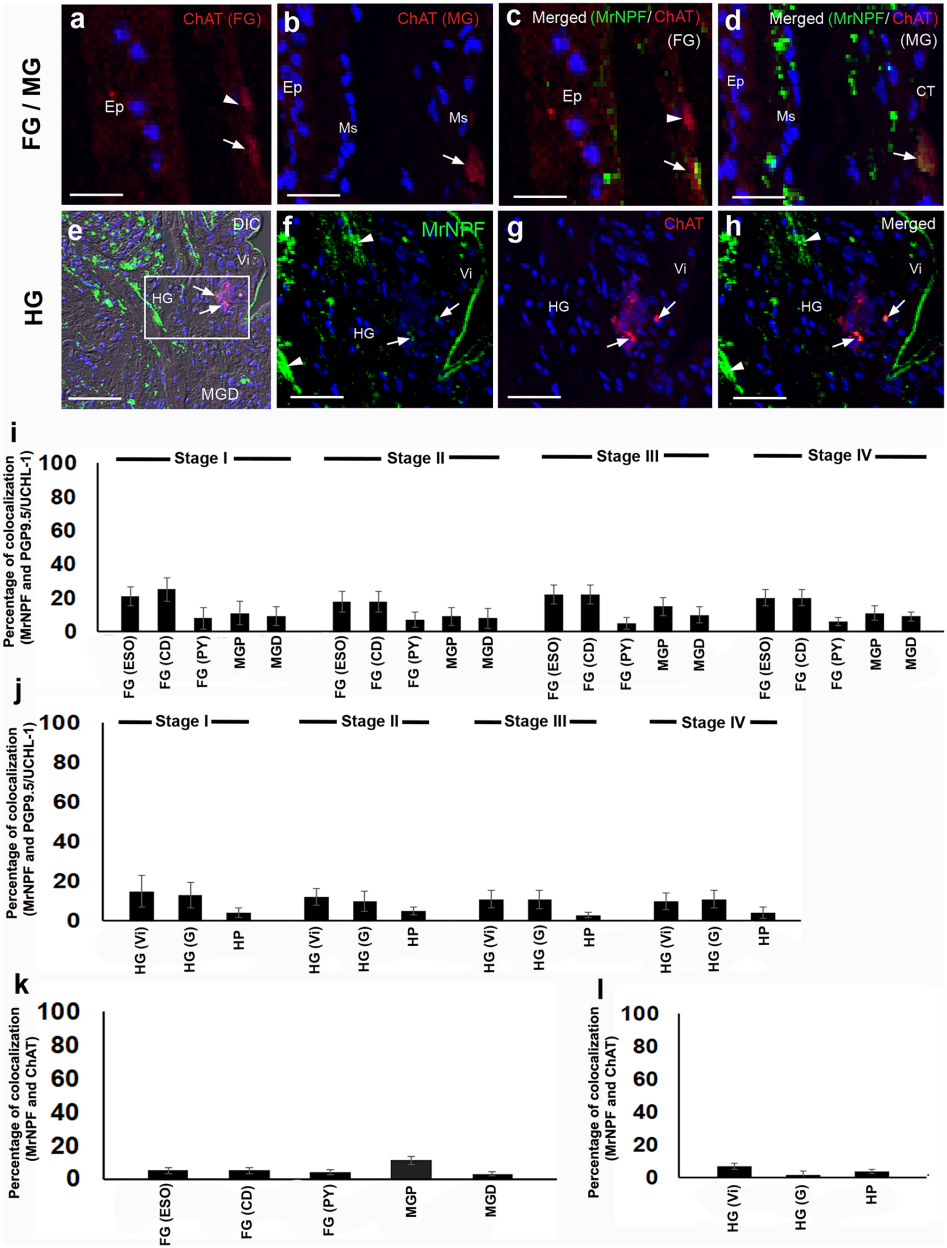
Representative images showing the expression and colocalization of MrNPF-ir (green) and ChAT-like-ir (red) immunolabelling in the FG/MG and HG of female *M. rosenbergii* (at least *n* = 15 female prawns). The nuclei were counterstained with DAPI (blue). **a**, **b** ChAT-like-ir are expressed in a few cells of FG and MG (white arrows and arrowhead), respectively. **c**, **d** Colocalization of MrNPF-ir and ChAT-like-ir in a few cells of FG and MG (white arrows), respectively. The figure **e** is the HG section which is merged with DIC image. **f**, **g** Medium magnification images demonstrating both MrNPF-ir (**f**) and ChAT-like-ir (**g**) in HGCs (white arrowheads). **h** Colocalization of MrNPF-ir and ChAT-like-ir underneath the Vi part of HG (white arrows). **i**, **j** Histograms showing the percentage of colocalization of MrNPF-ir and PGP9.5/UCHL-1 in the FG (ESO, CD, and PY), MGP and MGD (**i**), HG (Vi and G) and HP (**j**) during the ovarian cycle. **k**, **l** Histograms representing the percentage of colocalization of MrNPF-ir and ChAT-like-ir in the FG (ESO, CD, and PY), MGP and MGD (**k**), HG (Vi and G) and HP (**l**) during the ovarian cycle. CT, connective tissue; Ep, epithelial cell; HG, hindgut; MGD, midgut distal part; Ms, muscle; Vi, villi. Scale bars 100 µm (**e**), 50 µm (**f**, **g**, and **h**), and 25 µm (**a**, **b**, **c**, and **d**)

We performed double immunolabelling for MrNPF with PGP 9.5/UCHL-1 or ChAT to determine if these MrNPF-ir cells were neurons or endocrine cells that could control the activity of digestive cells in female *M. rosenbergii*. In the Lp area of the FG, colocalization of MrNPF and PGP 9.5/UCHL-1 varicosities was found in the PY region (Fig. 8a–d). In the MG, there was colocalization of both MrNPF-ir and PGP 9.5/UCHL-1-like-ir in some groups of cells beneath the circular Ms layer (Fig. 8e–l). We found that the HG part had a few strong PGP9.5/UCHL-1-like-ir cells, but a few cells in the Vi part showed little colocalization of MrNPF-ir and PGP9.5/UCHL-1-like-ir (Fig. 8m–o). In the G part, strong MrNPF-ir and a few PGP 9.5/UCHL-1-like-ir cells were seen (Fig. 8p and q). Nevertheless, very few cells with positive colocalization of MrNPF-ir and PGP9.5/UCHL-1-like-ir were found in this gut region (Fig. 8r). In the HP, little colocalization of MrNPF-ir and PGP9.5/UCHL-1-like-ir was found, and a few colocalized cells are observed (Fig. 8s, inset). A small number of cells located below the Ms layer exhibited significant ChAT-like-ir in the FG and MG, respectively (Fig. 9a and b). MrNPF-ir and ChAT-like-ir were colocalized in a few cells underneath the Ms layer in the FG and MG (Fig. 9c and d). The Vi portion of the HG also showed considerable MrNPF-ir (Fig. 9e and f), but the proximal portions of the HG showed a few strong ChAT-like-ir cells (Fig. 9g). However, only few cells with positive colocalization were detected in this area (Fig. 9e and h).

**Fig. 10:**
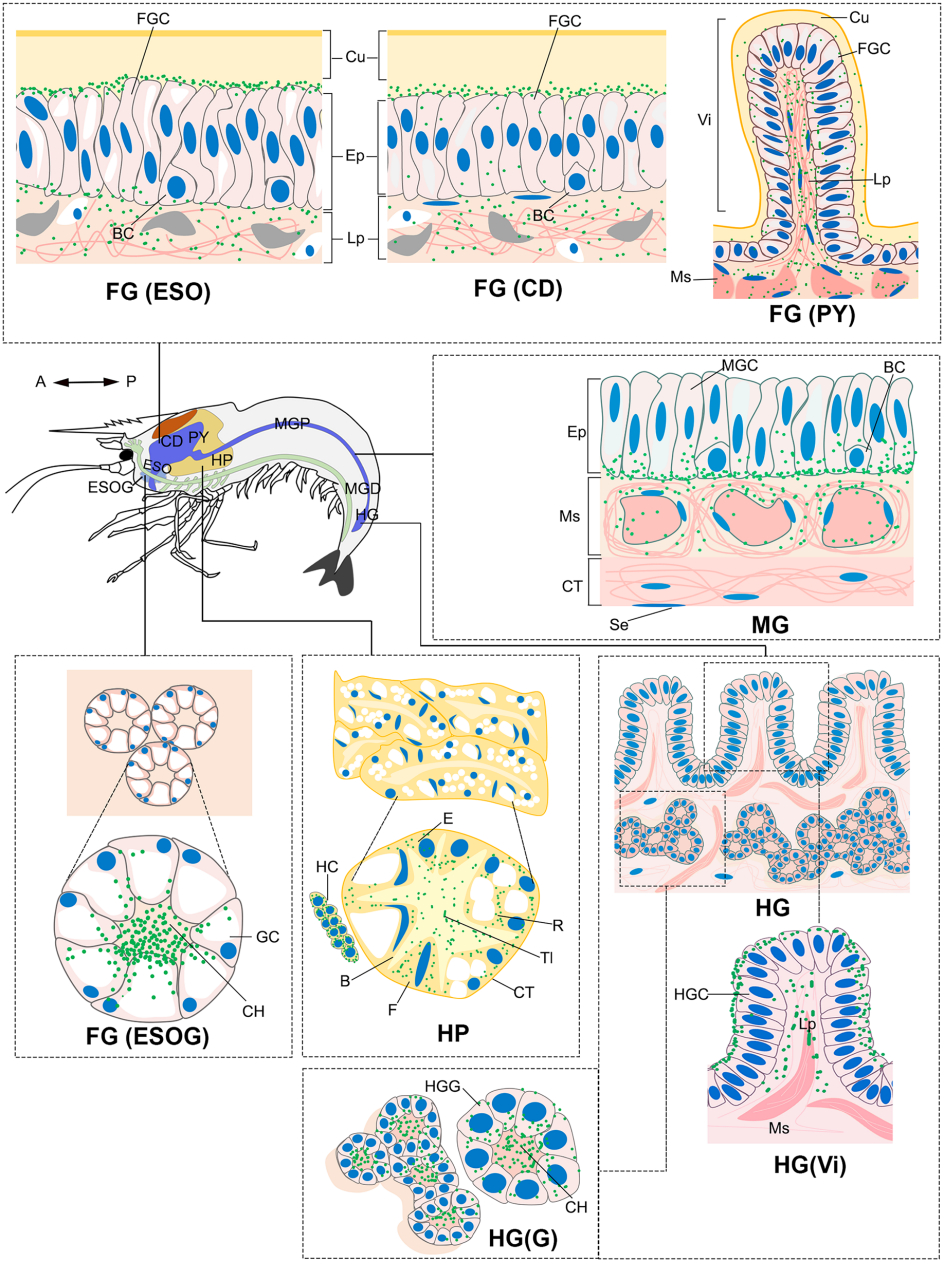
Schematic diagram illustrating the differential expression and distribution of MrNPF-ir (green dots) in the digestive organs of female prawn, *M. rosenbergii*. The dark green color represents strong MrNPF-ir, while the light green color shows weaker immunoreactivity. B, blister/vesicular cells; BC, basal cell; Bm, basement membrane; CD, cardia; CH, central lumen; CT, connective tissue; Cu, cuticle; E, embryonic cells; Ep, epithelium; ESO, esophagus; ESOG, esophageal gland; F, fibrillar cells; FG, foregut; FGC, foregut cell; GC, gland cell; HC, hemocyte; HG, hindgut; HGC, hindgut cell; HGG, hindgut gland; HP, hepatopancreases; Lp, lamina propria; MG, midgut; MGC, midgut cells; MGD, midgut distal part; MGP, midgut proximal part; Ms, muscle; PY, pylorus; R, resorptive cells; Se, serosa; Tl, tubular lumen; Vi, villi

There was little colocalization between the MrNPF and these two neuronal markers in digestive areas. In the ESO, CD, and PY of FG, the percentage of MrNPF-ir and PGP9.5/UCHL-1-like-ir colocalization during four ovarian stages were approximately 20%, 25%, and 10%, respectively (Fig. 9i and j), whereas the percentage of MrNPF-ir and ChAT-like-ir colocalization were around 7%, respectively, in the same digestive regions (Fig. 9k and l). In the MGP and MGD regions, only 13% and 8% of MrNPF-ir and PGP9.5/UCHL-1-like-ir colocalization were found (Fig. 9i), while about 12% and 5% of MrNPF-ir and ChAT-like-ir colocalization were detected in the MGP and MGD areas (Fig. 9k). The HG showed a higher number of cells with MrNPF-ir and PGP9.5/UCHL-1-like-ir colocalization, with around 20% of the cell population (Fig. 9j), whereas MrNPF-ir and ChAT-like-ir showed colocalization rates of about 10% and 5% in the Vi and G regions, respectively (Fig. 9l). The presence and distribution of MrNPF-ir in the digestive organs during the ovarian cycle are summarized and illustrated in Fig. 10. The dark green color represents strong MrNPF-ir, while the light green color indicates weaker immunoreactivity. Strong MrNPF-immunoreactivities were observed in the Ep, Ms, and Lp of the ESO, ESOG, CD, and PY. The MG cells and the Ms layer exhibited intense MrNPF-ir. Additionally, MrNPF-ir was detected in the Ep of the villi and gland regions in the HG. Notably, MrNPF-ir was also more intense in the F-, R-, and B-cells in the HP (Fig. 10).

## Discussion

In the present study, we have shown that MrNPF was expressed in all digestive organs of female *M. rosenbergii*, and its expression levels changed during various stages of the ovarian cycle. By and large, MrNPF mRNA expression levels in most digestive parts gradually increased from stage I to higher level in stages II and III, then declined in stage IV. Additionally, there was a wide spread distribution of MrNPF in several regions, including epithelium, muscular layer, and lamina propria. Interestingly, there were few colocalization between MrNPF and PGP9.5/UCHL-1 or ChAT, at these sites, suggesting that most of the expression occurred in non-neuronal cells, but possibly in digestive tract-associated endocrine cells, muscle, and epithelial cells. Furthermore, our findings suggest that MrNPF expression in digestive tissues might be associated with the ovarian cycle and indicate a possible important link of MrNPF’s role in feeding and ovarian maturation in this female prawn.

### Expression and distribution of MrNPF-ir in the FG

In female *M. rosenbergii*, the differential expression of MrNPF-ir were observed in the Ep layer and Ms of the ESO part of the FG. There have been reports regarding the expression and roles of NPF and FMRFamide-like peptide in the digestive organs in platyhelminthes, insects, and some crustacean species (Mertens et al. 2002; Lu and Pietrantonio 2011; Bao et al. 2015; Fadda et al. 2019). In platyhelminthes, numerous NPF-ir positive nerves were found prevalently in the muscle of the pharynx or ESO (Day and Maule 1999). In cestode, *Moniezia expansa*, NPF-ir was detected in the muscular sucker and ESO, suggesting NPF may be involved in delivering food to the intestine and expelling waste (Maule et al. 1992; Day and Maule 1999). In turbellarians, NPF-ir cells and fibers were detected in a muscular pharynx, indicating the role of NPF in the regulation of digestive processes (Day and Maule 1999). In insect species, FMRFamide has been reported to be linked to a variety of digestive processes, including gut muscle contraction (Schoofs et al. 1993), salivary gland secretion (Duve et al. 1992), food intake (Gonzalez and Orchard 2009), and the release of digestive enzymes in the MG (Nachman et al. 1997). In mosquito larvae *Aedes aegypti*, Aedae-NPF could decrease the transepithelial voltage across the anterior stomach, implicating the modulatory role of NPF in ion transport. In the eastern subterranean termite, *Reticulitermes flavipes*, NPF-ir axons were also observed in the ESO, suggesting that NPF may be involved in allowing and pumping food through the gut (Nuss et al. 2008). In spider crab, *Maja brachydactyla*, the ESO is responsible for transporting food from the mouth and opening to the stomach (Castejón et al. 2018). The Ep of the ESO is the simple columnar epithelium coated by a cuticle. The Ep contains numerous microtubules which may help to maintain and modulate cell structure and shape during expansive and contractive activities of the ESO during food swallowing (Mykles 1979; Castejón et al. 2018). Similar event occurs in *M. brachydactyla*, as the peristaltic movements of the ESO during food swallowing cause extrinsic muscles to push the Ep toward the connective tissue, allowing the lumen to expand (Castejón et al. 2018). Thus, the presence of MrNPF in the Ep layer and Ms of ESO in female *M. rosenbergii* could suggest a modulatory role in ESO expansion and contraction during the food-swallowing processes.

Strong MrNPF-ir was found in the Ep and Lp of the CD and PY of female *M. rosenbergii*. Aedae-NPF was also found to regulate and coordinate the anterior stomach functions, including alkalization and motility (Onken et al. 2004). In decapod crustaceans, the food in the CD is physically broken down and combined with the digestive fluid, while the chymus is filtered through a cuticular filter system in the PY, and the filtrate is further transferred into the HP for nutritional absorption (Vogt 1996; Štrus et al. 2019). In female *M. rosenbergii*, the expression of MrNPF-ir in the Ep and Lp of the CD and PY could suggest that MrNPF has some function related to the mixing and chemical breakdown of the food. However, this hypothesis needs further study.

In the ESOG, strong MrNPF-ir was found in the G cells and CC in female *M. rosenbergii*. In other decapod crustaceans, the ESOG or tegmental gland can be found close to the mouthparts and play an important role in helping the passage and digestion of food. In *M. brachydactyla*, this gland is comparable to salivary glands, as it has acid substances and mucous that could aid in digestion processes, to lubricate the luminal surface and facilitate food passage (Hunt et al. 1992; Castejón et al. 2018). In the lobster, *H. americanus*, tegmental glands produce a waxy secretory substance, and release acid mucopolysaccharides to lubricate the mouthparts (Felgenhauer 1992; Smolowitz 2021). Thus, the strong MrNPF-ir presence in the ESOG of *M. rosenbergii* may suggest its role in modulating the secretion of lubricant and possible digestive substances to help lubricate the mouth part and the preliminary digestion of food.

### The existence and differential expression of MrNPF-ir in the MG

Strong MrNPF-ir was detected in the MGCs, BL, and varicose fibers in the muscular layer of the MG in female *M*. *rosenbergii.* In insects, numerous enteroendocrine cells (EECs) that express one or more peptide hormones are assumed to be the source of the neuroendocrine factors that regulate digestive activities. NPF released by the EECs in the MG may activate receptive axons on the gut surface, bridging the gap between the gut and nervous system via the brain-gut axis (Nuss et al. 2008). In termite *R. flavipes*, NPF-ir fibers was present in the MG cells, implying that NPF may permit or control the pumping of food from the stomach (Nuss et al. 2008). In locust *S*. *gregaria*, the expression of *NPF* transcript was found in the MG, implying the role of NPF in controlling the MG activities (Stanek et al. 2002; Gonzalez and Orchard 2008; Nuss et al. 2008). In silk moth *Bombyx mori* larvae, MG showed the highest expression of *Bom-NPFR* transcripts, and *NPF* mRNA was upregulated during the start of feeding, suggesting that the MG may be a key target of NPF (Yamanaka et al. 2008). In corn earworm *Helicoverpa zea* and other insects, EECs in the MG may act as a sensory epithelium and such cells effectively serve as a sensory element of a gustatory signaling pathway. In fruit fly *D. melanogaster*, both *NPF* transcripts and NPF-ir were found in the CNS and MG, suggesting its roles in feeding, digestion, and providing important link between the brain and gut axis (Brown et al. 1999; Fadda et al. 2019). NPF expression was observed in the proximal and middle parts of the MG in *D. melanogaster* (Veenstra et al. 2008; Nässel 2018; Hung et al. 2020; Yoshinari et al. 2021). In *D. melanogaster*, dispersed EECs in the MG are likely the most plentiful source of NPF (Brown et al. 1999; Veenstra et al. 2008; Veenstra 2009), and NPF was found to be the only RFamide expressed in EECs in the MG (Chintapalli et al. 2007; Veenstra et al. 2008). NPF-ir was found in EECs of the *Drosophila* MG, where it was colocalized with neuropeptide-like precursor 2, tachykinin, and orcokinin, suggesting an important role of NPF as incretin-like EEC-derived hormones to modulate glucagon and insulin production, and/or to mediate sugar-dependent metabolism via glucagon- and insulin-like hormones (Hung et al. 2020; Yoshinari et al. 2021; Kruangkum et al. 2022). Additionally, peptides produced by EECs in the MG have been thought to be involved in the regulation of trypsin synthesis and release, as well as ion and water balance (Barillas-Mury et al.^1995^; Winther and Nässel 2001; Christie et al. 2007). However, morphology of EECs in *M. rosenbergii* have not yet been characterized and described. Thus, specific markers and/or ultrastructural morphology are needed to identify EECs in the MG of this prawn species.

In addition to the expression and roles of NPF in insects, there have been a few reports on the presence of NPF and FMRFamide in digestive organs in decapod crustaceans. In the mud crab *Scylla olivacea*, the expression of NPF-II-ir was observed only in the intestine, but not detected in the stomach and HP (Kruangkum et al. 2022). By contrast, NPF-II-ir was found in the intestine, stomach, and HP in *S. paramamosain* (Bao et al. 2015), implying that this NPF isoform might have a species-specific function. In male prawn, *M. rosenbergii*, *NPF* mRNA in the MG was detected at a high level by RT-PCR, suggesting its important role in controlling feeding (Thongrod et al. 2017). As well, in *L. vannamei* and *M. marginatus*, *NPF* transcripts were detected in the MG tissue using RT-PCR, which implies its control over MG activities,concerning nutrients, ion, and water absorption by the tissue and possibly also the release of other peptide hormones (Christie et al. 2011). In the present study, the prevalence of MrNPF-ir in the MGCs and varicose fibers of the muscular layer of the MG suggesting that MrNPF may be involved in the regulation of trypsin synthesis and release as well as ion and water homeostasis, as reported earlier (Winther and Nässel 2001; Christie et al. 2007).

### The presence and differential expression of MrNPF-ir in the HG

The expression and distribution of MrNPF-ir were detected in the Ep of Vi and G regions of the HG in female *M. rosenbergii*. There have been reports regarding the expression of NPF and/or FMRFamide in insects and crustaceans. In the 5^th^ instar *R. prolixus*, the presence of NPF-like-ir processes was seen in the HG, indicating a potential neuroregulatory function of NPF in this organ (Gonzalez and Orchard 2008; 2009). In *Drosophila melanogaster*, high doses of its NPF (DrmNPF) and *Anopheles gambiae* NPF (AngNPF) had little impact on the K+ influx, but strong effect on myoinhibitory function (Gonzalez and Orchard 2008). In the barnacle, *Balanus amphitrite*, FMRFamide-like-ir (FLI-ir) in varicose nerve endings in the HG were observed near the circular Ms cells, implicating that FLI-ir may act as a neurotransmitter or a neuromodulator that regulates the movement of the material toward the HG (Gallus et al. 2006). In the lobster, *Homarus americanus*, the sixth abdominal ganglion contains cells whose axons project to the HG via the intestinal nerves to innervate and coordinate contractions of the HG during defecation (Winlow and Laverack 1972). Later, in the same lobster species, FLI-ir was observed in a cluster of cells in the posterior region of A6 ganglion, suggesting functional roles for FLI in controlling the HG motility (Kobierski et al. 1987). In the crayfish *Procambarus clarkii*, the expression of FLI was observed in the intestinal nerve, which localizes in the sixth abdominal ganglion (A6) and innervates the HG. Thus, it is possible that the release of FLI coordinates HG movement (Mercier et al. 1991). In female *M. rosenbergii*, we found significant MrNPF-ir in the Ep and HGG of the HG, implying that MrNPF may be important in regulating HG motility and coordinating defecation through the anus. Previously, we also found the presence of strong MrNPF-ir in neurons and fibers within the A6 abdominal ganglion of female *M. rosenbergii* (Tinikul et al. 2022b). Thus, it is possible that MrNPF fibers from the A6 ganglion may innervate and control activities of the HG. However, further studies are needed to determine which exact nerve fibers controlling the HG activities in female *M. rosenbergii*.

### Expression and distribution of MrNPF-ir in the HP

In the present study, it was found that F- and B-cells of the HP displayed strong MrNPF-ir with the highest numbers of cells at the late ovarian stages, and the lowest numbers at early stages. Reports on NPF or FMRFamide expression in the HP of decapod crustaceans are scarce. Hence, we discussed the expression and distribution of NPF in related to their possible related functions in the HP. In decapod crustaceans, the HP cells are involved in absorption, nutrient assimilation, waste excretion, and immune response (Ruiz et al. 2019, 2020; Vogt 2019). In *Macrobrachium carcinus* and *M. rosenbergii*, the middle region of the HP tubules is primarily made up of F- and/or B-cells, indicating that these cells may be involved in intense secretion of digestive enzymes for extracellular digestion as well as some intracellular digestion (Sonakowska et al. 2015). F-cells are main secreting cells that produce digestive enzymes, including amylase, trypsin, chitinase, and cellulase (Lehnert and Johnson 2002). Besides, B-cells may help with intracellular digestion and nutrient absorption (Al-Mohanna and Nott 1989; Vogt 2019). The cytoplasm of B-cells has many small vacuoles and a few acid glycoproteins, which are thought to be involved in digestion (Ribeiro et al. 2014; Ruiz et al. 2019). Additionally, the HP is a major source of immune reactive molecules, including lectins, nitric oxide (NO), stress proteins, antibacterial and antiviral proteins, enzymes, and apoptotic genes. In male prawn *M. rosenbergii*, MrNPF in the HP could modulate autophagy to help balance energy expenditure and energy intake during starvation (Thongrod et al. 2018). Our finding that MrNPF-ir was found in F- and B-cells suggests the role of this neuropeptide in modulating intracellular digestion, releasing digestive enzymes, and regulating immune responses in female *M. rosenbergii*. Additionally, the increase in the number of immunoreactive B-cells following the ovarian cycle may be involved in higher activity of digestion and nutrient absorption to gain high energy for vitellogenesis at the late ovarian stage in this crustacean species.

In addition to F- and B-cells, MrNPF-ir was also detected in the R-cells, with their numbers appearing to be two times higher than the F- and B-cells during the ovarian cycle. R-cells are the most noticeable cell type in the HP epithelium of decapod crustaceans, where they are characterized by the presence of a large numbers of irregularly shaped lipid-filled vacuoles and glycogen deposits in the cytoplasm (Icely and Nott 1992; Vogt 2019). These cells are important in the uptake and storage of nutrients in crustaceans (Vogt 1996; Hu and Leung 2007). R-cells were also suggested to be involved in synthesizing lipoprotein in *M. nipponense* (Han et al. 1994), as well as being the site of Vg synthesis in *M. rosenbergii* (Jasmani et al. 2004). In the present study, we showed strong expression and highest numbers of MrNPF-ir R-cells. Our earlier report supports this finding since MrNPF was able to promote vitellogenesis in female *M. rosenbergii* (Tinikul et al. 2017). Hence, it is possible that MrNPF may be involved in modulating the production of vitellogenin by R-cells during ovarian maturation in female *M. rosenbergii*. In addition, we noticed the number of MrNPF-ir in R-cells slightly decreased during the late ovarian stage. This was supported by a slight decrease in the expression of MrNPF levels in the ovary at the late ovarian stages of female *M. rosenbergii* (Tinikul et al. 2017). In *M. rosenbergii*, HP is the main source for Vg synthesis, as Vg is secreted into hemolymph and accumulated into the ovary (Lee and Chang 1999; Tsukimura 2001). Thus, it is possible that the expression of MrNPF-ir in R-cells of HP may be correlated with MrNPF-ir in the ovary during the late stage. However, this needs to be ascertained by further studies.

Finally, we used antibodies against PGP 9.5/UCHL-1 or ChAT to colocalize with MrNPF in order to prove whether MrNPF-producing cells in the digestive tract of female *M. rosenbergii* were neurons. We found very few cell-like spots of colocalization between the MrNPF and these two neuronal markers in all parts of the digestive tract. Some cells are stained with the pan-neuronal marker but did not colocalize with anti-ChAT. Additionally, we did not observe the characteristics of neurons, including processes extending from neurons, around these MrNPF-ir positive structures. Recently, it was found that there were endocrine cells in the digestive tract that exhibit both endocrine and neuron-like characteristics (Kontogeorgos 2018). These endocrine cells have specialized sensory microvilli that project into the gut lumen, and they respond to luminal stimuli by releasing their hormones into the Lp (Furness et al. 1999; El-Salhy 2009); thus, the hormones released in the Lp could act locally on neighboring cells via a paracrine mode, or on cells in the nearby parts of the digestive tract via circulating blood through an endocrine mode (El-Salhy et al. 2017). In insects and crustaceans, endocrine cells in the digestive tract have been shown to act via paracrine mode, such that the peptides released locally also influence nearby digestive cells, as well as having an impact on distant target organs (Fujita et al. 1981; Veenstra and Lambrou 1995). In *L. vannamei* and *Melicertus marginatus*, it was suggested that NPF acts as a local paracrine factor in the MG that directly controls nutrient, ion, and water absorption, as well as indirectly regulating the release of other peptide or hormones from this tissue (Christie et al. 2011). In crabs and spiny lobsters, some stomach muscles are depolarized by glutamate and others are depolarized by acetylcholine (Marder 1974, 1976; Atwood et al. 1977). Neurons innervating stomach muscles have been shown to contain ChAT (Marder 1976). In spiny lobsters, some muscles respond to both glutamate and acetylcholine (Lingle 1980). This information probably explains why some cells showed pan-neuronal marker staining, but they did not show anti-ChAT colocalization in female *M. rosenbergii*. Taken together, we believe that most of the MrNPF-ir positive cells might not be neurons, but could be some kind of digestive tract-associated endocrine cells in female *M. rosenbergii.*

Remarkably, the higher MrNPF mRNA levels found at stages II and III of the ovarian cycle were observed in nearly all parts of the digestive system. This suggests that the MrNPF may assist in maintaining high food intake during the proliferative and premature stages of ovarian development since increased food intake contributes to growth and reproductive success, both of which require high energy reserves. Mature female prawns must consume substantial amounts of nutrients through feeding in order to build up the raw materials and energy needed to sustain body processes as well as for vitellogenesis and ovarian maturation. It is interesting that the pattern of MrNPF expression levels in the digestive organs here also coincides with those found in the brain, subesophageal, and thoracic ganglia (Tinikul et al. 2022b), which are all linked to the ovarian cycle in female *M. rosenbergii*. Thus, our findings strongly suggest that MrNPF acts as a link in the CNS-gut axis that controls the feeding strategy to sustain healthy status for female prawns and subserve ovarian maturation, leading to the reproductive success of this important crustacean species.

In conclusion, the present study provides novel findings on the expression and distribution of MrNPF in several parts of the digestive organs during the ovarian cycle in female *M. rosenbergii*. Our study also offers important new insights into the hormonal control of feeding and ovarian maturation of this female prawn.

## Abbreviations

Ab: Abdominal part
AG: Abdominal ganglia
AN: Antennae
B: Blister/vesicular cell
BC: Basal cell
Bm: Basement membrane
BR: Brain
CD: Cardia
Cep: Cephalothorax
CH: Central lumen
Cr: Crypt
CT: Connective tissue
Cu: Cuticle
E: Embryonic cell
Ep: Epithelium
ES: Eyestalk
ESO: Esophagus
ESOG: Esophageal gland
F: Fibrillar cell
FG: Foregut
FGC: Foregut cell
GC: Gland cell
HC: Hemocyte
HG: Hindgut
HGC: Hindgut cell
HGG: Hindgut gland cell
HP: Hepatopancreas
Lp: Laminar propia
Lu: Lumen
MG: Midgut
MGC: Midgut cell
MGD: Midgut (distal part)
MGP: Midgut (proximal part)
Ms: Muscle
OV: Ovary
PY: Pylorus
R: Resorptive cells
RT: Rostum
Se: Serosa
TG: Thoracic ganglia
Tl: Tubular lumen
Vi: Villi
